# Supplementary figures and images for: On the Impact of the Pangenome and Annotation Discrepancies While Building Protein Sequence Databases for Bacteria Proteogenomics
Source: Front Microbiol. 2019 Jun 20;10:1410. doi: 10.3389/fmicb.2019.01410 (PMC6596428; doi:10.3389/fmicb.2019.01410)

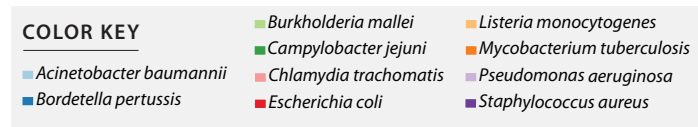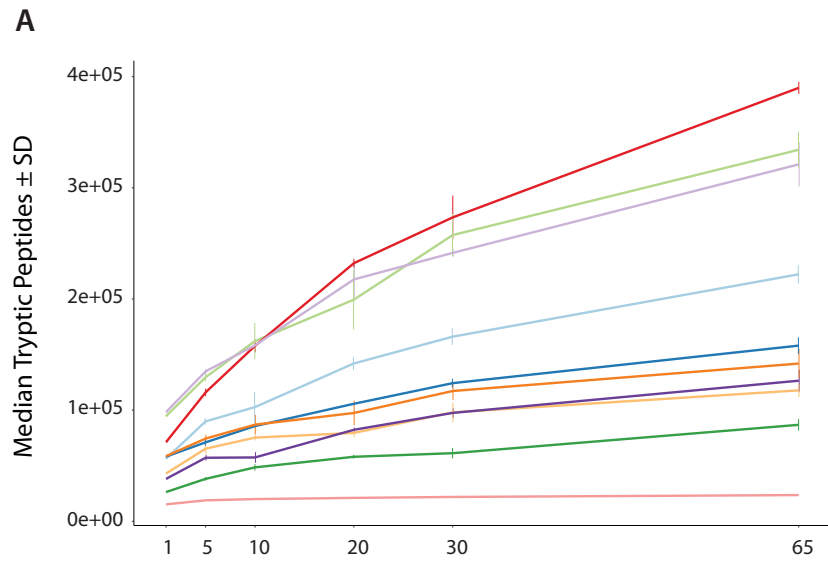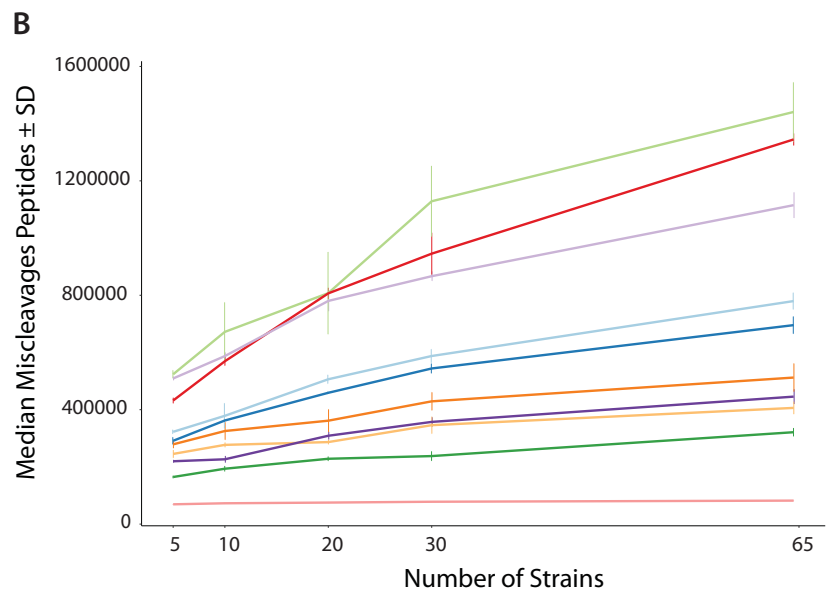

Supplement: FIGURE S1 — Number of tryptic peptides per MSMSpdbb database. In (A) only fully tryptic peptides are considered, while in (B) peptides with up to two miscleavages are also counted, a standard search parameter choice in proteomics. Values in x axis show the number of strains used in the database. All values plotted in the graph are given in Supplementary Table S1. [file Image_1.pdf]

A

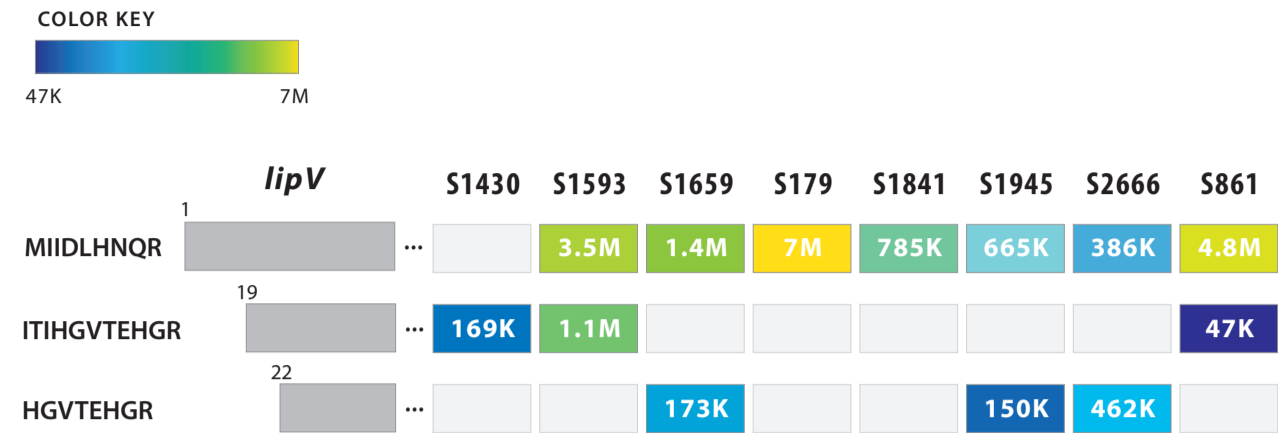

B

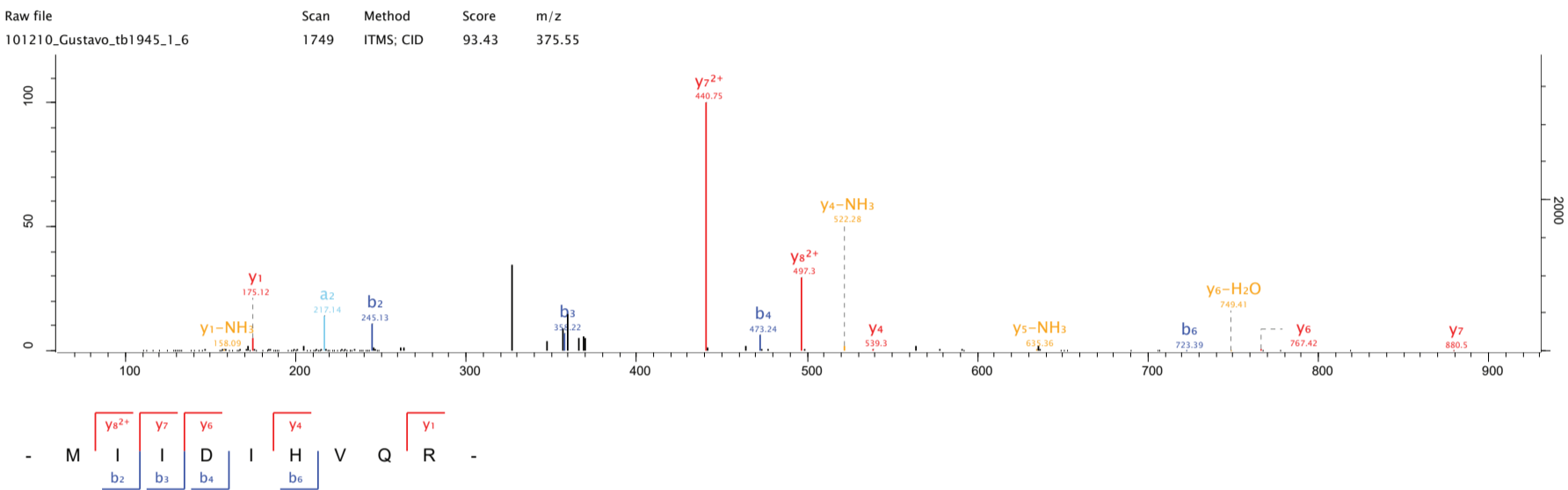

C

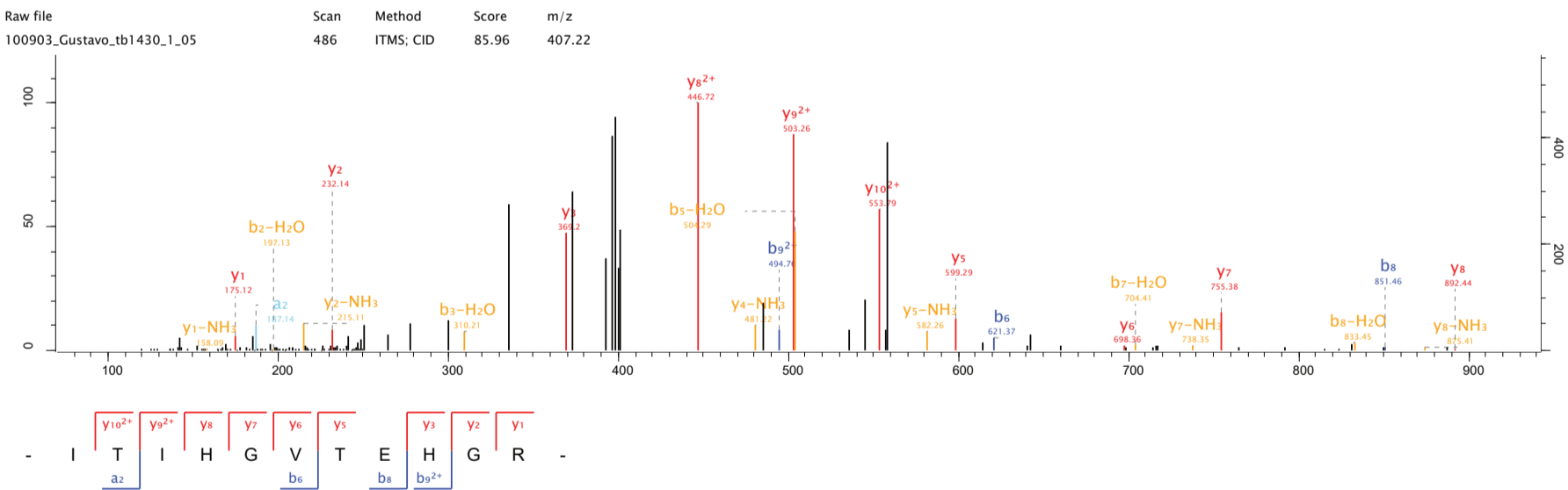

D

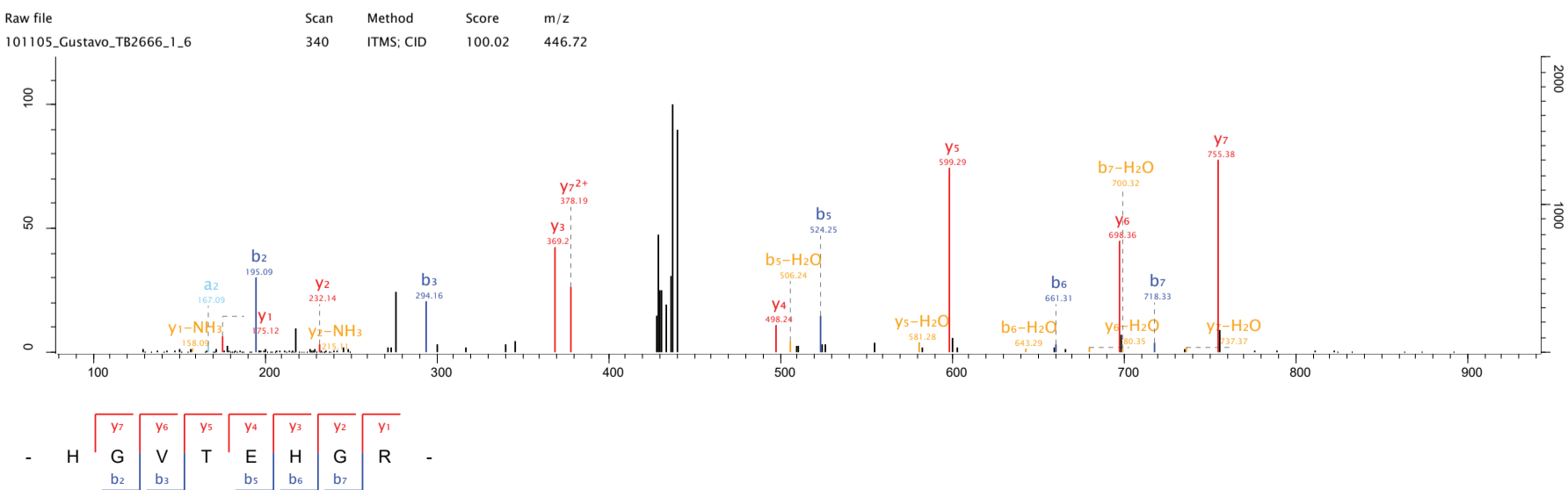

Supplement: FIGURE S2 — Score distribution in subpopulations of identified peptides. All identified peptides in S. aureus (A) and Mtb (B) datasets were divided according to their occurrence across all strains. For Mtb they were divided as peptides annotated in more than 33 strains, annotated in 13 to 32 strains, or in 12 strains or less. For S. aureus the values for similar groups is 100, 21 to 99 and 20 strains or less, respectively. Score medians in all valid identifications ranged from 121.5 to 138.2, while decoy identification had median scores of 59.2 in Mtb and 51.3 in S. aureus (red lines). [file Image_2.pdf]

**A**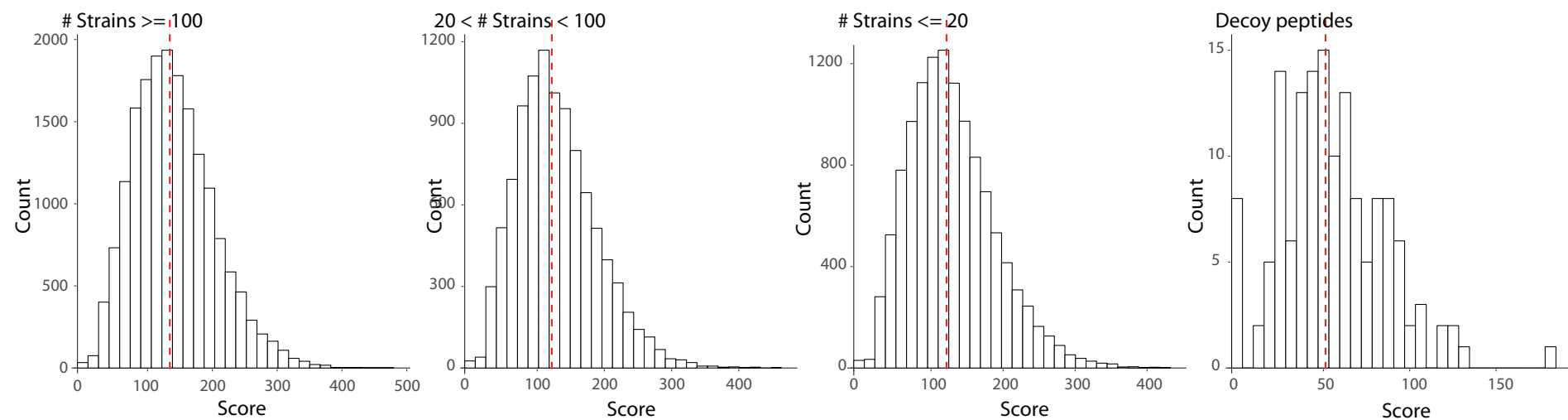**B**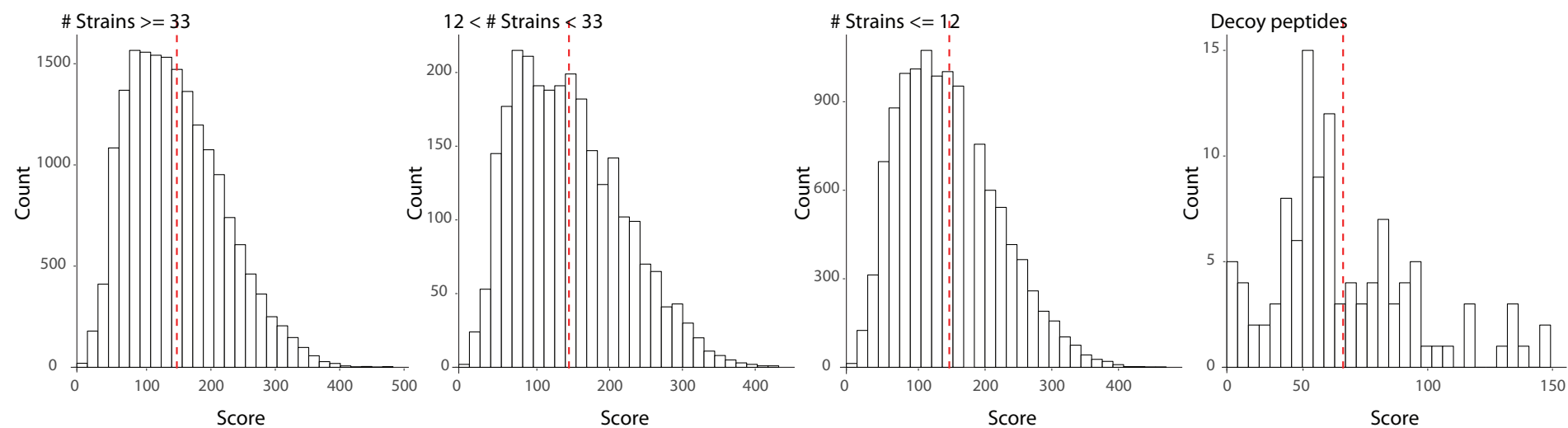

Supplement: FIGURE S3 — Multiple TSS identified in protptionn lipV. (A) Three possible TSS choices were identified in clinical Mtb strains. The most upstream prediction (MIIDLHNQR) is the most abundant variant observed in all strains, except strain S1430 which has only the variant with TSS at position 19 (ITIHGVTEHGR) of the reference sequence, and strain S2666 which TSS at position 22 (HGVTEHGR) is predominant. Two possible TSS choice variants were also observed at high levels for strain S1593. (B–D) MS2 spectra for all three peptides mentioned above. [file Image_3.pdf]

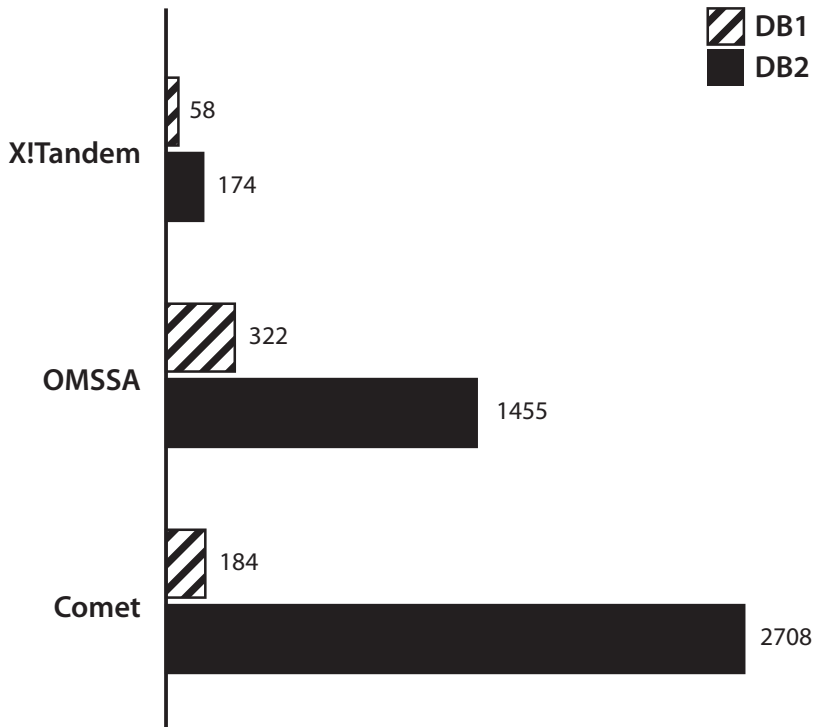

Supplement: FIGURE S4 — Analysis time for peptide search engines other than Andromeda/MaxQuant. Bars shows, in minutes, the time spent for peptide identification using X!Tandem, OMSSA or Comet, using either the reduced DB1 or the concatenated DB2 databases. [file Image_4.pdf]
