# Supplementary material for: On the Impact of the Pangenome and Annotation Discrepancies While Building Protein Sequence Databases for Bacteria Proteogenomics
Source: Front Microbiol. 2019 Jun 20;10:1410. doi: 10.3389/fmicb.2019.01410 (PMC6596428; doi:10.3389/fmicb.2019.01410)
Supplement: Supplementary file 6 [file Data_Sheet_1.docx]

**SUPPLEMENTARY TEXT**

**Log file format, database format and rationale**

All fasta files should be saved in same folder before the script all_fasta.pl will be executed. This script will select the fasta with higher number of protein entries as Query (called Query 1 in Figure 1A), and perform a BLAST sequence alignment between the query and all entries in each of the remaining fasta files (called Subjects in Figure 1A). This alignment is Query to Subject direction. Parameters used to define a true alignment are given in Methods. Once this is finished, the script will then perform a Subject to Query alignment, selecting each Subject fasta file and aligning it to Query 1. In a bidirectional best hit approach, a homologous is defined if the best sequence pair selected in one direction is also the best pair selected in the other direction.

For example (proteins in red are present in Subject 1 fasta file):

1) When all entries in Query 1 are aligned to all entries in the first Subject (here called Subject 1), **protein A** from Query 1 aligns to the following proteins from Subject 1: **protein X** (score 2872, best hit); protein Y (score 872); and protein Z (score 456). When all proteins from Subject 1 are submitted to alignment against Query 1, protein X aligned to: **protein A** (score 2872, best hit), protein B (score 1542), protein C (score 1002). Therefore, since **protein X** is the best alignment possible for **protein A**, and protein A is the best alignment possible to protein X (protein A ↔ protein X), those are defined as homologues.

2) Similarly as above, **protein A** from Query 1 best hit is protein X in Subject 1. But in this example protein X aligns to following Query 1 proteins: protein D (score 3211, best hit), protein A (score 2872) and protein B (score 1542). Since best alignment for protein X was not protein A (protein A → protein X, protein X → protein D), proteins A and X will not be considered homologous. If protein D best hit is also protein X, protein D and protein X will be considered homologous.

This first bidirectional alignment round will divide all entries from Query 1 into two groups, which will be saved in two distinctive temporary files (Figure 1B): entries from Query 1 which found a homologue in one or more of the Subjects are copied into a file named Homologues. All homologues from different subjects that were associated to **protein A** from above example are clustered into a single group and further analyzed together (script pep_trip.pl, see below). Entries in Query 1 which did not found a homologue are considered ‘uniquely annotated’ and present only in Query 1. Uniquely annotated entries are not further processed, and later the script pep_trip.pl will copy them straight into the final database (Figure 1D).

The all.fasta.pl script will now have completed all homologue discoveries regarding Query 1. Before a new Query is selected, the following modifications will be performed: the strain selected for Query 1 will be removed from the dataset, since all its protein entries were already pair wise aligned and classified. The remaining fasta files (Subjects in Figure 1A) will be significantly reduced to save computational power in the following rounds. Any protein entry in a Subject fasta which was classified as a homologue for Query 1 will be removed from the respective Subject fasta. For example, if Subject 1 contained 4,100 protein entries, and from those 3,000 entries were classified as homologue of a Query 1 entry, for the next round Subject 1 will be reduced from 4,100 to 1,100 protein entries. This will be applied to all Subject files.

The following BHH round is a repetition of the previous round. Query 1 is removed from the dataset; the remaining Subjects are reduced, and one is then selected as a new Query (Query 2 in Figure 1E) using same parameter as before (file with higher number of entries); BLAST BHH will be performed with Query 2 as anchor. When done, as for the previous round two files will be created, one containing the uniquely annotated proteins only detected in Query 2, and one with homologous proteins from all strains. Note that the homologues identified on this round are all new associations which were not classified as homologues to any Query 1 protein in the previous round. When the two files are created, the round is finished. Query 2 is removed from dataset, remaining subject strains will have protein entries already classified as homologue of a protein in Query 2 removed, and a new Query (Query 3) will be selected for a new round. This will repeat until one of two conditions are met: a) there is only one Subject fasta file left, so when it is selected as Query *N*, there is no other fasta file to align it to, and all its entries will be classified as “uniquely annotated”; or b) there are more than one strain left as Subject, but all of their proteins were already classified as homologue of a previous Query fasta. The script will then terminate.

At this point, the user will have a folder containing several files divided in two groups: those that contain entries uniquely annotated in Query 1, Query 2, etc, and those files with all homologue associations from each round. The user will then run the pep_trip.pl script. As stated above, this script will not handle any of the uniquely annotated entries; those will be directly copied into the final database as they are seen in the original fasta files. But each homologue cluster in each “Homologues” file will be then treated as follow: first, the longest entry (i.e., higher number of amino acids in its sequence) is chosen as a reference sequence, to be used for further homologues comparisons. In the vast majority of cases, the longest variant represents the one with the further upstream translational start site predicted. We chose this parameter to select a reference sequence because it would be simpler to compare N-terminal peptides from shorter protein versions (i.e., different TSS choices) against the longest homologue. Once a reference is chosen, all other homologues in the cluster which are 100% identical are annotated. Below an example of this in the log file also created by pep_trip.pl:

# Accession Number KT00165

# Reference entry: Mycobacterium tuberculosis CCDC5180 [AEJ50946.1]

# Number of strains in homologue file = 63

# Number of strains with 100% identity = 34

# Fields: Entries with 100% identity

Mycobacterium tuberculosis Erdman (ATCC35801) [BAL66322.1]|Mycobacterium tuberculosis EAI5 [AGQ35613.1]|Mycobacterium tuberculosis KIT87190 [AID05372.1]|Mycobacterium tuberculosis ZMC13-264 [AII90912.1]|Mycobacterium tuberculosis ZMC13-88 [AII94827.1]|Mycobacterium tuberculosis KZN 1435 [ACT24722.1]|Mycobacterium tuberculosis 96075 [AIQ04953.1]|Mycobacterium tuberculosis 96121 [AIQ08970.1]|Mycobacterium tuberculosis Kurono [BAQ06391.1]|Mycobacterium tuberculosis H37Rv; TMC 102 [AIR15070.1]|Mycobacterium tuberculosis Beijing-like [AJW49690.1]|Mycobacterium tuberculosis SCAID 187.0 [ALB19503.1]|Mycobacterium tuberculosis F1 [AMC42501.1]|Mycobacterium tuberculosis F28 [AMC46746.1]|Mycobacterium tuberculosis 2242 [AMC68877.1]|Mycobacterium tuberculosis 2279 [AMC73722.1]|Mycobacterium tuberculosis 22115 [AMC78103.1]|Mycobacterium tuberculosis 37004 [AMC82306.1]|Mycobacterium tuberculosis 22103 [AMC86510.1]|Mycobacterium tuberculosis 26105 [AMC90722.1]|Mycobacterium tuberculosis SCAID 320.0 [ANZ83020.1]|Mycobacterium tuberculosis SCAID 252.0 [AOE36714.1]|Mycobacterium tuberculosis 1458 [AOZ43556.1]|Mycobacterium tuberculosis Beijing-like/35049 [ARM94742.1]|Mycobacterium tuberculosis Beijing-like/36918 [ARM98774.1]|Mycobacterium tuberculosis Beijing-like/38774 [ARN02798.1]|Mycobacterium tuberculosis Beijing/391 [ARN06790.1]|Mycobacterium tuberculosis Beijing-like/50148 [ARN10805.1]|Mycobacterium tuberculosis Beijing-like/1104 [ARN14833.1]|Mycobacterium tuberculosis NCGM946K2 [BAW13347.1]|Mycobacterium tuberculosis HN-024 [BAX27999.1]|Mycobacterium tuberculosis HN-205 [BAX41444.1]|Mycobacterium tuberculosis HN-321 [BAX45518.1]|Mycobacterium tuberculosis HN-506 [BAX49573.1]|

In the example above, user can see that the cluster contained 63 homologues, and since Mtb used data from 65 strains, two strains did not have this homologue present or annotated. The reference protein is observed with 100% identity in 34 of those 63 strains. In the final database the protein is represented with the given accession number (KT00165). Three tryptic peptides were colored red below to help visualize polymorphs present in remaining homologues (next paragraph):

>KT00165 hypothetical protein CCDC5180_2109 [Mycobacterium tuberculosis CCDC5180]

MVKPAARLSVVVGDVAANYDGRVVV**APTGQAVDVAVR**EGAGDVGYSVER**ENLPADDPVR**NGNRWRVIAVDTEHHRIAARRLGDGARAAFSGDYLHEHITHGYAITVHASQGTTAHSTHAVLGDNTSR**ATLYVAMTPAR**ESNTAYLCERTAGEGARVDLAGWDLWVSGKAEAMSDEKSASPVWCRVGARCDHRGKRSCW

For the remaining homologues which are not 100% identical, the script will find which polymorphisms exists in each of their sequences. For this, the reference protein is *in silico* digested with trypsin, not allowing any miscleavages. Each of the remaining homologues are treated the same, and their tryptic peptides are compared to reference tryptic peptides. Identical tryptic peptides are not considered, and peptides that are not identical to reference protein are annotated. The annotation includes the type of modification observed, for example a single amino acid polymorphism (SAP), a different N-terminal prediction (TSS), a SAP involving Arg or Lys which then deletes or creates a new tryptic site (SAP R|K), etc. Some peptides are classified as NC (NOT FOUND) because their sequence does not show any resemblance to reference peptides. They are most probably frame shits caused by indels. All those variant peptides will only be considered in the final database if their amino acid length is between 7 and 35 residues. They are reported in the log as follow:

# Accession Number KT00166

# Artificial entry

# Fields: modification, peptide, peptide mutated, amino acid substitution, strain

SAP R|K **ATLYVAMTPAR** ATLYVAMTPASESNTAYLCER R->S Mycobacterium tuberculosis Beijing [AOT73993.1]|

# Accession Number KT00167

# Artificial entry

# Fields: modification, peptide, peptide mutated, amino acid mutation, strain

SAP **ENLPADDPVR** ENLPPDDPVR A->P

Mycobacterium tuberculosis CCDC5079 [AGM00879.1]|Mycobacterium tuberculosis CCDC5079 [AEJ47327.1]|

# Accession Number KT00168

# Artificial entry

# Fields: modification, peptide, peptide mutated, amino acid mutation, strain

TSS - MAPTGQAVDVAVR - Mycobacterium tuberculosis CDC1551 [AAK46666.1]|Mycobacterium tuberculosis H37Ra; ATCC 25177 [ABQ74099.1]|Mycobacterium tuberculosis RGTB327 [AFE17165.1]|Mycobacterium tuberculosis RGTB423 [AFE13516.1]|Mycobacterium tuberculosis H37Rv [AFN50273.1]|Mycobacterium tuberculosis 7199-99 [CCG12189.1]|Mycobacterium tuberculosis Beijing/NITR203 [AGJ68397.1]|Mycobacterium tuberculosis EAI5/NITR206 [AGL31804.1]|Mycobacterium tuberculosis F11 [ABR06664.1]|Mycobacterium tuberculosis CCDC5079 [AGM00879.1]|Mycobacterium tuberculosis HKBS1 [AHJ43089.1]|Mycobacterium tuberculosis BT2 [AHJ47236.1]|Mycobacterium tuberculosis BT1 [AHJ51383.1]|Mycobacterium tuberculosis CCDC5180 [AHJ55530.1]|Mycobacterium tuberculosis K [AIB48974.1]|Mycobacterium tuberculosis 49-02 [CDM10666.1]|Mycobacterium tuberculosis H37RvSiena [AJF03671.1]|Mycobacterium tuberculosis Haarlem [EBA42651.2]|Mycobacterium tuberculosis KZN 4207 [AEB03807.1]|Mycobacterium tuberculosis DK9897 [APR59438.1]|Mycobacterium tuberculosis MTB1 [ARF02123.1]|Mycobacterium tuberculosis MTB2 [ASD94342.1]|Mycobacterium tuberculosis KZN 605 [AFM49052.1]|Mycobacterium tuberculosis W-148 [EGE50860.1]|Mycobacterium tuberculosis H37Rv [CCP45098.1]|Mycobacterium tuberculosis CTRI-2 [AEN00768.1]|

In the database, each variant peptide is added to a single entry, as seen below;

>KT00166 hypothetical protein TM57_03665 [Mycobacterium tuberculosis Beijing]

ATLYVAMTPASESNTAYLCER

>KT00167 hypothetical protein CCDC5079_2137 [Mycobacterium tuberculosis CCDC5079]

ENLPPDDPVR

>KT00168 hypothetical protein MTCTRI2_2352 [Mycobacterium tuberculosis CTRI-2]

MAPTGQAVDVAVR

Once all homologue files are handled, pep_trip.pl will terminate. At the end, it will have created two files: a log file which describes all proteins added to the final database (if they come from a homologue cluster, detailed information is given as seen above), and the fasta database itself. Entries in the final database are created as follow: a) entries with protein sequences which did not gave results in the BHH analysis (called uniquely annotated); b) a “reference” entry, containing the longest protein version of each homologue cluster; and c) entries containing individual tryptic peptides classified as polymorphic. This database will be ready to be used in any peptide search engine as long as it allows the use of customized databases with non-standard fasta entry headers format (example of standard header format: Uniprot, Genbank, etc).
